# Hyponatremia and Identification of Seniors at Risk (ISAR) Score in Geriatric Patients: An Analytical Cross-Sectional Study

**DOI:** 10.7759/cureus.49493

**Published:** 2023-11-27

**Authors:** Rajarajeswari TB, Arul Senghor KA, Vinodhini V.M., Kumar JS, N Prasath

**Affiliations:** 1 Family Medicine, SRM Medical College Hospital and Research Centre, Kattankulathur, IND; 2 Biochemistry, SRM Medical College Hospital and Research Centre, Kattankulathur, IND; 3 Internal Medicine, SRM Medical College Hospital and Research Centre, Kattankulathur, IND

**Keywords:** cognitive function, length of stay, geriatrics, isar, hyponatremia

## Abstract

Aim and objective

Hyponatremia is the most common electrolyte abnormality in hospitalized patients. Age is an important, strong independent factor for hyponatremia. Geriatric at-risk groups are identified with six straightforward dichotomous questions. The research aims to determine the incidence of hyponatremia and correlate the identification of seniors at risk (ISAR) score with electrolytes in elderly individuals.

Materials and methods

The 334 geriatric population of elderly men and women were recruited based on age cut-off as more than 70 to 75, 76 to 80, and more than 80. Patients were categorized based on serum sodium levels as severe hyponatremia group A (< 125 mEq/L), moderate hyponatremia B (126 to 130 mEq/L), mild hyponatremia C (131 to 135 mEq/L) and normonatremia D (136 to 145 mEq/L). At the time of admission, the ISAR Tool was utilized to identify the participants with hyponatremia at risk. Electrolytes sodium, potassium, and chloride were analyzed in the Beckman Coulter AU480 autoanalyzer by indirect ISE method. The mean of the data was compared with ANOVA, and the ISAR risk score was correlated with electrolytes by Pearson correlation analysis; p < 0.5 is considered significant.

Results and discussion

About 16.76 % (56 patients) had severe hyponatremia (Serum sodium 119.14± 4.14 mEq/L) with a high ISAR score (6.0) and were reported as critical alert results by the central laboratory. Based on age-wise category, 70 to 75 yrs had serum sodium levels of 133.82 mEq/L, 76 to 80 yrs had 128.4 mEq/L, and more than 80 years had serum hyponatremia 119.17 mEq/L. Geriatrics with normonatremia had a 1.22 ISAR score, whereas geriatrics with severe hyponatremia had a 6.0 ISAR score. Correlation analysis of the ISAR score revealed a statistically significant negative correlation with sodium (r = - 0.53), potassium (r = - 0.34), and chloride (r = - 0.30) levels. Likewise, the length of hospital stay of the geriatrics with severe hyponatremia was prolonged (19 days), which contributed to the deterioration of health status; thereby, better healthcare follow-up is encouraged.

Conclusion

Hyponatremia is a critical alert analyte that is common in hospitalized geriatric patients. The ISAR score system points towards the individuals who are at risk and need immediate correction. Thereby, correction of sodium levels and close monitoring of the elderly patient enables quick recovery, and the consequences of hyponatremia are circumvented.

## Introduction

Hyponatremia is the most significant and frequent clinical issue in hospitalized patients. The types of hyponatremia are Euvolemic, hypovolemic, or hypervolemic hyponatremia. Sodium levels and volume status assessment are crucial components in properly diagnosing and managing hyponatremia. The degree of symptoms is contingent upon the rate, duration, and severity of hyponatremia. Cognitive impairment, abnormalities in gait, and a higher risk of falls and fractures are linked to hyponatremia in geriatric patients [1].

Hyponatremia is increasingly prevalent in the elderly due to decreased water and electrolyte balance caused by dietary and environmental factors. Clinico-etiological factors were evaluated in hospitalized patients with hyponatremia, and the mortality was 20.9% in geriatrics admitted to the intensive care unit [2]. The prevalence of hyponatremia seems to be quite alarming, which accounts for 37.5% of patients admitted for neurosurgery in the South Indian population [3]. Geriatric individuals with low sodium levels (131 mmol/L) have a greater risk of falls and fractures. Sodium levels have been linked to bone health, osteoclastic activity, and increased fragility of bone. Prolonged hyponatremia poses a significant risk, elevating the likelihood of geriatric syndrome development, falls, low bone density, fractures, cognitive decline, and all-cause mortality. It is associated with several detrimental outcomes in healthcare, involving an upsurge in mortality, an increase in hospital stay length, and frequency of admission in the intensive care unit [4].

"Hyponatremia is defined as a serum sodium level less than 135 mEq/L, mild chronic hyponatremia as a serum sodium level between 130 and 135 mEq/L, moderate hyponatremia as a sodium level between 125 and 129 mEq/L, and severe hyponatremia as a serum sodium level < 125 mEq/L" [5]. Age-related physiological changes that are inevitable make hyponatremia more likely. In addition to the altered synthesis of vital hormones, including arginine vasopressin, aldosterone, and atrial natriuretic peptide, these changes also include decreased glomerular filtration rate, decreased urine concentrating capacity, thirst sensitivity, and free water clearance.

An individual's sodium and water homeostasis is essential for adequate physiological function. However, disturbances due to body volume imbalance or disease pathologies such as vomiting, diarrhea, renal disease, and cardiovascular disorders associated with multi-system physiological dysregulation would result in hyponatremia. Adaptive behavioral and physiological responses are triggered to preserve body sodium and fluid levels. It's ideal for correcting the underlying pathology; thereby, sodium and total body water composition is maintained [6]. Hyponatremic high-risk patients are identified using the Identification of Seniors at Risk (ISAR) tool, which includes six straightforward dichotomous questions [7]. The research aims to determine the incidence of hyponatremia and the relative risk of ISAR score in elderly individuals.

## Materials and methods

The analytical cross-sectional study included geriatrics men and women in the age group greater than 70 years via selective sampling technique. The study was conducted at SRM Medical College Hospital and Research Center, General Medicine ward, from July to December 2022.

Sample size calculation based on the prevalence of hyponatremia (22%) in the geriatric population [8].

N = (z)2 p ( 1 - p ) / d2

Z (level of confidence according to the standard normal distribution) = 1.96; p (estimated proportion of the population) = 22% = 0.22 q = 1-p = 1-0.22 = 0.78 d (tolerated margin of error) = 5%, N = 264

Inclusion criteria

Elderly patients of either gender, aged above 70 years, visiting the study site with baseline sodium levels < 135 mEq/L, were included in this study. 

Exclusion criteria

Geriatric individuals with kidney diseases who underwent any brain surgery in a road traffic accident, with incomplete patient details, were excluded. The Declaration of Helsinki was followed during the study's performance. Protocols were followed as per the guidelines of Biomedical research in human participants. The institutional ethical committee approved this research work (SRMIEC-ST0722-014). All the participants were informed and explained about the significance of the investigation. The Patient's Consent Form approval was obtained before the commencement of work. The details of the participants were documented in case report form (CRF) i) The first section included patient details such as age, sex, demographics, UHID, IP/OP/IP-emergency, and the Patient circumstances at the time of admission ii) Biochemical investigations such as serum sodium, potassium, chloride, bicarbonate were analyzed in the automated Beckman Coulter AU480 with dedicated reagents by indirect ISE electrodes.

Based on serum sodium levels, the severity of the hyponatremia was categorized as severe hyponatremia less than 125 mEq/L, moderate hyponatremia 125 - 130 mEq/L and mild hyponatremia 131 - 135 mEq/L. ISAR tool was utilized to identify individuals with poor cognitive functional ability and risk of morbidity. The six-item self-report screening questionnaire with dichotomous yes/no responses is the basis of the ISAR tool that addresses functional cognitive decline, mortality, hospitalization, and adverse outcomes. ISAR score cut-off of 2 indicates a low-risk group with safe discharge of geriatric patients. ISAR maximum score of 6 indicates a very high-risk group that needs further assessment and follow-up of the hospitalized patient [7]. The length of stay of hospitalized geriatric patients, from admission to discharge, was also documented. 

Statistics

Data were examined using the statistical program for social services (SPSS 22.0) to compare quantitative variables. The difference in the parameter mean levels between the groups was analyzed using ANOVA. The ISAR risk score was correlated with electrolytes by Pearson correlation analysis. The data with a p-value less than 0.05 was considered statistically significant. 

## Results

In this study, 334 geriatric patients admitted to the General Medicine department of SRM Medical College Hospital were observed between July and December 2022. Among the admitted geriatrics, 65.56% (219 patients) were males and 34.43% (115 patients) were females. Severe hyponatremia was observed in 16.76% of hospitalized geriatric patients. Whereas 24.25% had moderate hyponatremia and 44.6% had mild hyponatremia. The identification of seniors at risk (ISAR) tool was found to be increased in those serum sodium levels that were found to decrease. Table 1 represents the severity of hyponatremia based on serum sodium cut-off concentration. Elderly individuals with severe hyponatremia had mean sodium levels of 119.14± 4.14 mEq/L.f

**Table 1:** Comparison of serum sodium levels in geriatric individuals ISAR – “Identification of Seniors at Risk”. The data with a p-value less than 0.05 was considered statistically significant. Values are expressed in Mean with standard deviation. NS stands for "not significant." ** Significant ***Extremely Significant

PARAMETERS	Group A Na <125 (mEq/L) n = 56	Group B Na125-130(mEq/L) n = 81	Group C Na 131-135 (mEq/L) n = 149	Group D Na136-145 (mEq/L) n = 48	F value	P value
Age (yrs)	79.57 ± 4.01	77.76 ± 3.6	75.32 ± 7.7	74.27 ± 7.15	9.25	< 0.001***
Sodium (mEq/L)	119.14± 4.14	128.22± 1.12	133.17 ± 1.63	139.5 ± 3.68	68.56	< 0.001***
Potassium (mEq/L)	4.95 ± 0.90	4.49 ± 0.85	4.08 ± 0.71	3.75 ± 0.63	27.07	< 0.001***
Chloride (mEq/L)	88.57 ± 7.3	91.25 ± 4.68	97.02 ± 13.2	104.06 ± 5.6	47.02	< 0.001***
Bicarbonate(mEq/L)	17.9 ± 4.78	21.13 ± 5.9	22.61 ± 8.01	23.5 ±5.5	8.16	< 0.001***
ISAR score	6 ± 0.0	4.79 ± 0.66	2.74 ± 0.48	1.22 ± 0.41	72.24	< 0.001***

ISAR score determines the risk a geriatric patient with hyponatremia can develop. Identification of Seniors at Risk components revealed that 54% of the geriatrics needed help regularly to take care of them. The most vital component is hospitalization; 53% of geriatrics patients were hospitalized during the past 6 months. Eyesight is another ISAR tool that revealed that 83% had vision problems and 54% had memory problems. Moreover, 59.5% of the geriatrics had been taking 3 different medications previous to current hospitalization.

**Figure 1:**
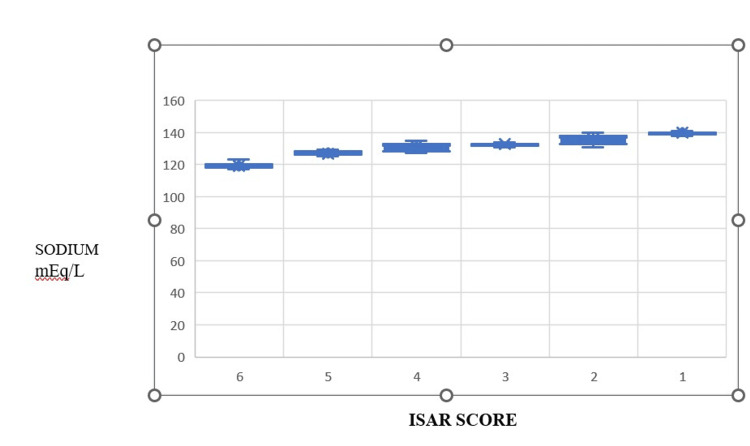
Correlation of ISAR score and sodium concentration ISAR: identification of seniors at risk

Table 2 represents the correlation analysis of the ISAR score with the electrolyte panel (Figure 1). It was observed that sodium revealed a statistically significant negative correlation, whereas potassium and chloride revealed a moderate negative correlation.

**Table 2:** Correlation coefficient analysis of ISAR score with electrolyte analytes Correlation coefficient scale: ± 1.0: Perfect correlation, ± .8 to 0.99: Very strong, 0.6 to 0.8: Strong correlation, ± 0.4 to 0.6: Moderate correlation, ± 0.2 to 0.4: weak, ± 0 to 0.2: very weak

PARAMETERS	r value	P value
Sodium (mEq/l)	- 0.503	< 0.001 *
Potassium (mEq/l)	- 0.34	0.006 *
Chloride (mEq/l)	- 0.30	0.003 *
Bicarbonate (mEq/l)	- 0.19	0.10 (NS)

**Figure 2:**
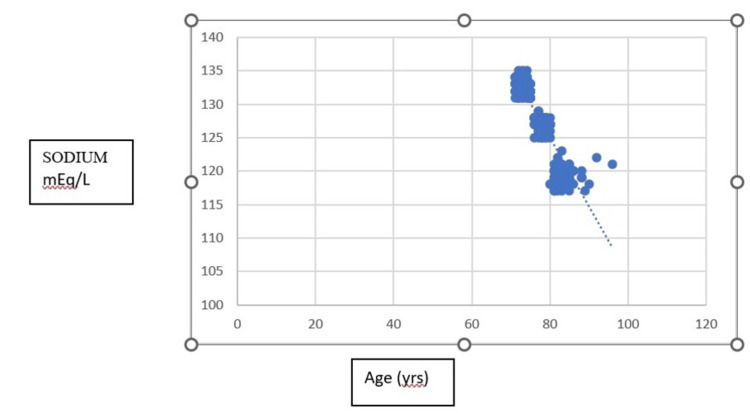
Comparison of serum sodium levels in age-wise category

Figure 2 depicts the concentration of serum sodium concentrations as per the age-wise category. It was found that geriatrics aged more than 80 yrs had a serum sodium concentration of 119.17 mEq/L; in the age group between 76 to 80 yrs and 71 to 75 yrs, it was observed that the serum sodium concentration of 128.4 mEq/L and 133.82 mEq/L respectively.

**Figure 3:**
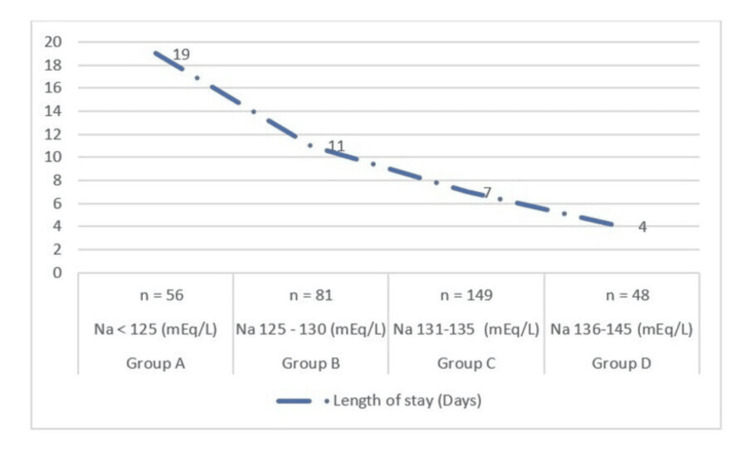
Comparison of length of hospital stay based on sodium concentrations

Figure 3 represents the sodium concentration with respect to the length of stay in the hospital. It was observed that geriatric patients with serum sodium concentration below 125 mEq/L had a length of stay of approximately 19 days compared to patients with normal sodium concentrations who had four days of hospital stay. 

## Discussion

Hyponatremia is the most common complication in hospitalized geriatric patients. Severe hyponatremia contributes to neurological issues like cognitive problems, posture, and gait abnormalities. Hyponatremia would result in prolonged hospitalization of the patients. The critical alert analyte sodium levels less than 125 mEq/L are severe hyponatremia. It's very important to categorize with the patient's age and ISAR score that provides the necessities to commence immediate treatment modalities. The elderly individuals with an ISAR score of 6 indicate a very high-risk group who need further assessment and follow-up of the hospitalized patient. This comprehensive geriatric assessment is ideal for identifying at-risk patients in chronic care settings. Kapoor et al. concluded that cognitive scores were less in the hyponatremic group as compared to normonatremic group [9].

ISAR screening tool

In this study, 334 geriatric patients were admitted to the General Medicine department. Geriatrics assessment was revealed as per the ISAR tool and found that 16.76% of participants had severe hyponatremia and were reported as critical alert results by the central laboratory. Moreover, as per the ISAR screening tool, 54% of the patients had poor memory and poor vision. Hyponatremia results in an osmotic insult, ending up in cerebral edema as a clinical consequence. 

Thus, in patients with severe hyponatremia, neurological symptoms, and volume status are essential to identify geriatric patients for immediate treatment as addressed by the Clinician. Treatment for hyponatremia is a challenge for elderly hospitalized patients who are getting intravenous fluids that are hypotonic or whose fluid intake is not being carefully regulated [10].

Age

According to age wise study on sodium levels, it was observed that patients older than 75 exhibited hyponatremia, which is in concordance with the researchers' findings [11,12]. The geriatric patients had severe hyponatremia and were found to be aged more than 80 years. Researchers had reported hyponatremia in geriatrics, and these patients' scores on all Geriatric Assessment tests were noticeably lower than those of the control group [13]. It's a fact that with increasing age, the individual's renal capacity to excrete water deteriorates, which is related to the reduction of the Glomerular filtration rate [14].

Length of stay

In this context, the geriatric patients with severe hyponatremia had increased length of stay compared to normonatremia. In our study, the patients with hyponatremia were found to be hospitalized for an average of 19 days, which in concomitant Baser et al. [14]. As expected, geriatric patients with severe hyponatremia had longer lengths of stay compared to moderate and mild hyponatremia. This is concomitant with the findings of Balan et al. [15].

This study is unique in utilizing the ISAR tool to assess geriatrics at risk, and constant monitoring is advisable on the ward side. The geriatrics with severe hyponatremia ISAR tool score was found to be high, and the decline in cognitive function reveals the prime importance of addressing immediately, thereby, any adverse health outcome and mortality [16,17].

It was demonstrated that, compared to geriatrics with normal serum sodium levels, the individuals with severe hyponatremia had poor geriatric assessment standards [18,19]. Also, Chavvaro et al. [20] have evaluated the predictive cut-off value more than 2 beyond, which is related to increased morbidity. It has been mandated that frequent monitoring of electrolyte levels is ideal for diagnosis and prognosis; thereby, prolonged length of stay at the hospital is minimized.

Age, length of stay, and ISAR score reflect the state of general functioning and balance as interpreted through geriatric assessment tools. The study's outcome supported the clinical utilization of self-report screening tools related to adverse health consequences with impaired cognitive function.

The study's limitations were that the geriatric assessment tool did not apply to ventilator support patients. Further follow-up of the patients after treatment was not done.

## Conclusions

Geriatric hospitalized patients with severe hyponatremia have ISAR risk scores and are subjected to prolonged lengths of stay. ISAR screening tool is ideal for early identification of elderly patients with hyponatremia. It's very important to identify elderly patients with hyponatremia and deliver precise management; thereby, morbidity is reduced. Thus, elderly patient with hyponatremia should be treated with an increased level of care in a multidisciplinary setting to improve their health outcomes and reduce morbidity.
